# Low-Computational-Cost Hybrid FEM-Analytical Induction Machine Model for the Diagnosis of Rotor Eccentricity, Based on Sparse Identification Techniques and Trigonometric Interpolation

**DOI:** 10.3390/s21216963

**Published:** 2021-10-20

**Authors:** Carla Terron-Santiago, Javier Martinez-Roman, Ruben Puche-Panadero, Angel Sapena-Bano

**Affiliations:** Institute for Energy Engineering, Universitat Politècnica de València, Camino de Vera s/n, 46022 Valencia, Spain; cartersa@etsii.upv.es (C.T.-S.); jmroman@die.upv.es (J.M.-R.); rupucpa@die.upv.es (R.P.-P.)

**Keywords:** fault diagnosis, sparse identification, model order reduction, induction machines

## Abstract

Since it is not efficient to physically study many machine failures, models of faulty induction machines (IMs) have attracted a rising interest. These models must be accurate enough to include fault effects and must be computed with relatively low resources to reproduce different fault scenarios. Moreover, they should run in real time to develop online condition-monitoring (CM) systems. Hybrid finite element method (FEM)-analytical models have been recently proposed for fault diagnosis purposes since they keep good accuracy, which is widely accepted, and they can run in real-time simulators. However, these models still require the full simulation of the FEM model to compute the parameters of the analytical model for each faulty scenario with its corresponding computing needs. To address these drawbacks (large computing power and memory resources requirements) this paper proposes sparse identification techniques in combination with the trigonometric interpolation polynomial for the computation of IM model parameters. The proposed model keeps accuracy similar to a FEM model at a much lower computational effort, which could contribute to the development and to the testing of condition-monitoring systems. This approach has been applied to develop an IM model under static eccentricity conditions, but this may extend to other fault types.

## 1. Introduction

One of the most common electrical machines in industry is induction machines (IMs). These machines play an important role in the safe and efficient operation of various types of industrial applications due to their numerous strengths, such as simplicity, ruggedness and high reliability, at relatively low cost. However, they are not free from faults that may lead to unexpected failures, causing large economic losses. For this reason, reducing operation and maintenance costs as well as improving reliability have become crucial issues in their maintenance [1]. Much has been proposed in the technical literature for condition-monitoring (CM) systems, the stator current being one of the most widely used due to its low requirements. Not much hardware (only a current clamp is needed) and low software resources are required [2]. Therefore, motor current signature analysis (MCSA) approach for IM fault diagnosis has become one of the most common and well-stablished method presently.

Online CM systems and CM systems based on artificial intelligence (AI), as neural networks (ANN) [3], principal component analysis [4] and more recently support vector machines (SVM) [5], are becoming very important in IMs CM since they greatly improve reliability and maintainability in a wide range of industrial applications. The development of these systems would allow the detection of faults at an early stage and to evaluate their evolution to define not only the maintenance operations but also the right time to implement them. As an example, knowing the right time to carry out maintenance tasks would help keeping the profitability of a wind farm along its operational lifetime [6].

IMs in many applications scenarios work under non-stationary conditions or even transient conditions, such as in wind farms, where the wind variability involves non-stationary working conditions. Under these conditions, traditional fast Fourier transform (FFT)-based techniques are no long effective to the fault diagnosis of the IM. Several advanced signal processing techniques have already been investigated to overcome the limitations of the conventional FFT-based techniques [7]. Nonetheless, the use of these complex techniques results in a large volume of data and its analysis requires high skilled maintenance professionals.

In an attempt to avoid these problems and to make diagnostic algorithms more reliable, artificial intelligence (AI) tools are used for fault diagnosis of the electrical rotating machines with the aim of detecting faults at very early stage and of reducing false alarms rates. These techniques could also be used with online CM systems, which continuously monitor the machine status. However, the development and training of these expert systems to predict the upcoming failure from IMs is a challenge presently, since it requires the obtaining of many current measurements from different types of machines and with different severity degrees of a given fault or simultaneous faults under different working conditions [8]. These requirements are difficult to replicate with actual IMs working in the industry, since there is a limited number of IMs running under faulty conditions. On the other hand, the use of IMs in laboratory test benches is costly; much destructive testing is needed, and the artificial introduction of progressive failure degrees and varying working conditions is challenging. Moreover, the condition-monitoring systems could be only tested with the machines available in the test benches and with the working conditions the test bench can reproduce. Therefore, test benches are a somehow limited source of data for the development and training of CM based on AI.

Alternatively, the interest in accurate models of faulty IMs is increasing. Models would allow the reduction of the number of destructive tests needed to develop new diagnostic techniques and to develop and test CM based on embedded devices [9]. They would also be very helpful to train expert systems [10], to develop vector classifiers [11], to obtain a comprehensive understanding of the observed phenomena and to define and compare different fault indexes [12]. These models, therefore, should consider the detailed structure of the machine to obtain simulation results that faithfully reproduce the behavior of the actual IM [13]. Moreover, they must allow the monitoring of the magnitude analyzed for fault detection, as well as running in real time to develop and test online condition-monitoring systems [14]. In the following subsection, the most recent advances in the development of faulty IM models are reviewed.

### Fault Modeling Methods

Several models of faulty IMs can be found in the technical literature, which can be broadly categorized as models based on electrical circuits, models based on magnetic circuits, models based on numerical methods and hybrid models. Models based on multiple coupled circuit (MCC) comprise the stator and rotor in multiple inductive circuits, which are coupled together [15]. Resistance parameters are usually estimated by analyzing the dimensions of conducting paths. However, the calculation of the inductance parameters of a faulty machine is far more complex. Several methods have been reported, winding function approach (WFA) and modified winding function approach (MWFA) being some of the most common methods to compute the inductance parameters [16,17].

Afterwards, with the aim of simplifying MCC models, d-q models were introduced. They use orthogonal components of voltage and currents by Park and Clark transforms [18]. The same parameter estimation techniques are typically used as in MCC models. They are very fast and, thus, can be implemented in hardware in the loop (HIL) simulation systems [19]. However, the applicability of these kind of models for fault diagnosis purposes is quite limited since they are not able to reproduce the effect of spatial harmonics or asymmetries, which directly affect variables such as speed, currents and other performance IM parameters required for CM systems.

Contrary to MCC approach, which is based on coupled electrical circuits, magnetic equivalent circuit (MEC) is based on detailed magnetic modeling of the machine by estimation of reluctances and permeances [20]. These models have the advantage of moderate computational complexity if compared to high-accuracy modeling such as finite element method (FEM), but the accuracy during transient conditions is usually limited, since these models do not usually include distributes circuit effects in the rotor conductor or the stator ring leakage inductance [21].

Accurate simulations of faulty IMs requires taking into account the non-linearities of the magnetic materials as well as avoiding the simplifying hypotheses regarding the geometry and windings arrangement. Circuit-based models run fast but cannot provide comprehensive modeling as numerical methods, as those based on FEM. FEM methods use the exact magnetic and geometric characteristics of the machine to compute their magnetic field distribution, thus, accurately reproduce the IM behavior under failure conditions. However, they require a significant computational capacity and long simulation times, which can vary from minutes to weeks or even months in cases of highly asymmetrical faults such as eccentricity or rotor broken bars [22]. The effects of these type of faults in electromagnetic parameters such as voltage, speed, torque, flux density and flux distribution for a faulty machine are accurately represented through time stepping finite element method (TSFEM) [23,24]. However, even with modern processors, the computational effort required to complete FEM evaluation is notable [25]. Differences of more than 3 h for a FEM analysis versus 7.6 s via an analytical method have been reported in [26]. On the other hand, fault conditions often imply that the simplifications commonly used to reduce computational costs and increase simulation speed, such as machine symmetry, cannot be used [27]. Thus, the study of several degrees of failure using FEM is a complex matter [28]. Moreover, running these models in hardware simulators, which allow reducing these times, is problematic so far.

To overcome these drawbacks, hybrid FEM-analytical model simulations have been recently proposed in the technical literature for fault diagnosis purposes [29]. FEM analysis is used to identify the parameters of an analytical model of the machine. The high accuracy of the model obtained can run in real time in HIL systems. However, the study of different degrees of a given fault or combination of several types of fault could be unaffordable, since it requires the full simulation of the FEM model for each scenario. To address these drawbacks, ref. [30] proposes the sparse subspace learning (SSL) and hierarchical Lagrange interpolation (HLI) polynomial from a selected number of FEM simulations to compute the inductance matrix of the faulty IM model. This method reduces the number of FEM simulations to obtain the coupling parameters of the faulty machine for each fault and severity degree under study, resulting in large savings in memory resources when compared with FEM. However, it still requires several GB in memory resources for every degree of a given fault. Therefore, although it reduces computational costs compared to traditional FEM methods, it still needs a large number of fully FEM simulations. Moreover, its implementation for other kind of machines or the inclusion of different kind of faults is challenging. In a similar way, ref. [31] presents an analytical model where FEM is used to compute the coupling parameters of the faulty machine. In this case, FEM analysis run on multiple processor cores working in parallel with each other to reduce the simulation time needs. Despite the improvements these approaches present, they still require long simulation times and large computational resources. Savings on these issues are essential where many studies are required, such as fault diagnosis testing. In this context, this paper proposes the use of the sparse identification and trigonometric interpolation polynomial to minimize the number of FEM simulations required to develop a hybrid FEM-analytical model of a faulty IM. A very reduced set of magneto-static FEM simulations is required to build the trigonometric polynomial basis with which compute the inductance matrix of a faulty machine. The proposed method is applied to develop an accurate model, valid to run in real time, that simulates various static eccentricity fault scenarios. The resulting model keeps a good accuracy while drastically reducing computational effort and simulation times.

The paper is structured as follows. In Section 2 the equations that define the analytical model of an IM are described and the process to calculate the coupling parameters is introduced. In addition, the characteristics of the case of study and the main drawbacks of the approach are shown. Section 3 presents the methodology followed to calculate the parametric basis. The main results are in Section 4, where the coupling parameters obtained with the proposed method are compared to the those obtained using FEM simulations. In Section 5 the fault diagnosis results are analyzed in detail. Finally, experimental results and conclusions are presented in Section 6 and Section 7 respectively.

## 2. System Equations

The behavior of an IM with m stator and n rotor phases can be defined by the following equations system [32,33]:

(1) $$\left[U_{s}\right]=\left[R_{s}\right]\left[I_{s}\right]+d\left[\Psi_{s}\right]/dt$$

(2) $$\left[U_{r}\right]=\left[R_{r}\right]\left[I_{r}\right]+d\left[\Psi_{r}\right]/dt$$

Where subscripts *s* and *r* are used for the stator and rotor, respectively. [U] is the phase voltage matrix, [I] is the phase current matrix, [Ψ] is the flux linkages matrix and [R] is the resistances matrix. $\left[R_{s}\right]$ is the submatrix of resistances for every stator phase and $\left[R_{r}\right]$ is the submatrix of resistances of every rotor phase in the case of wound rotor, or of every bar in the case of squirrel-cage rotor. [U] and [Ψ] are composed of:

(3) $$\left[U_{s}\right]={\left[u_{s1},u_{s2},...,U_{sm}\right]}^{T}$$

(4) $$\left[U_{r}\right]={\left[u_{r1},u_{r2},...,U_{rn}\right]}^{T}$$

(5) $$\left[\Psi_{s}\right]=\left[L_{ss}\right]\left[I_{s}\right]+\left[L_{sr}\right]\left[I_{r}\right]$$

(6) $$\left[\Psi_{r}\right]={\left[L_{sr}\right]}^{T}\left[I_{s}\right]+\left[L_{rr}\right]\left[I_{r}\right]$$

On the other hand, the electromechanical torque generated by the machine, $T_{e}$, is given by:

(7) $$\left[T_{e}\right]=\frac{1}{2}{\left[I\right]}^{T}\frac{d[L]}{d\theta }\left[I\right]$$

where θ is the mechanical or geometric angle between the main rotor axis and the stator fixed reference and [L] is the inductance matrix, which is given by:

(8) $$\left[L\right]=\left[\begin{array}{cc} L_{ss} & L_{sr}\\ L_{sr}^{T} & L_{rr} \end{array}\right]$$

where $\left[L_{ss}\right]$ contains the mutual inductances between the stator phases and their leakage inductances, $\left[L_{rr}\right]$ are the mutual rotor inductances between rotor phases and their leakage inductances and $\left[L_{sr}\right]$ contains the mutual inductances between stator and rotor phases. Finally, the mechanical behavior is modeled by the following equation:

(9) $$\left[T_{e}\right]-\left[T_{load}\right]=J\frac{d^{2}\theta }{dt^{2}}+B\frac{d\theta }{dt}$$

where $T_{load}$ is the mechanical load torque, *J* is the inertia moment and *B* is the friction coefficient.

**Figure 1:**
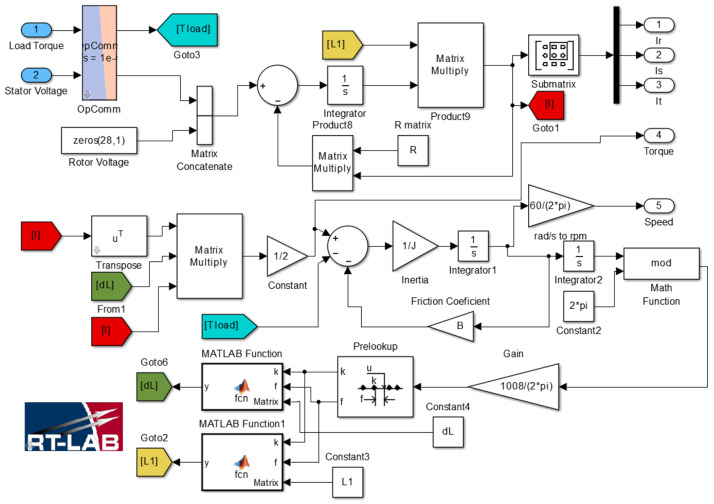
Analytical model of the induction machine using Equations (10)–(12) in Matlab/Simulink. The characteristics of the machine are found in Table 1.

In this work, the system of Equations (1)–(9) is solved using Matlab/Simulink. Thus, a model in Simulink has been developed for obtaining its numerical solution in the time domain, as shown in Figure 1. To this end, stator and rotor items are grouped in terms of differential equations as it shown in the following:

(10) $$\left[\begin{array}{c} U_{s}\\ U_{r} \end{array}\right]=\left[\begin{array}{cc} R_{s} & 0\\ 0 & R_{r} \end{array}\right]\left[\begin{array}{c} I_{s}\\ I_{r} \end{array}\right]+\frac{d}{dt}\left(\left[\begin{array}{cc} L_{ss} & L_{sr}\\ L_{sr}^{T} & L_{rr} \end{array}\right]\left[\begin{array}{c} I_{s}\\ I_{r} \end{array}\right]\right)$$

(11) $$T_{e}=\frac{1}{2}\left[\begin{array}{cc} I_{s} & I_{r} \end{array}\right]\frac{d}{d\theta }\left(\left[\begin{array}{cc} L_{ss} & L_{sr}\\ L_{sr}^{T} & L_{rr} \end{array}\right]\right)\left[\begin{array}{c} I_{s}\\ I_{r} \end{array}\right]$$

(12) $$T_{e}-T_{load}=J\frac{d^{2}\theta }{dt^{2}}+B\frac{d\theta }{dt}$$

Due to the presence of the derivatives in the Expressions (1), (2) and (7), the inductance matrix components $\left[L_{ss}\right]$, $\left[L_{rr}\right]$ and $\left[L_{sr}\right]$, also known as coupling parameters, must be computed with high accuracy and for every rotor position, especially if different fault conditions are to be detected and reliably diagnosed. This would require many FEM simulations, with their corresponding long simulation times and memory requirements, which this work tries to reduce using an approach based on the sparse identification and trigonometric interpolation polynomial. The computed coupling parameters are used in the analytical model, which can run in real time in a HIL system. Moreover, the user can modify in real time different parameters, such as the stator voltages, the frequency and the load torque. Thus, the model allows simulation of the machine under a wide range of working conditions and degrees of fault severity, which is one of the main requirements for the development of CM systems.

## 3. Proposed Method for Computing the Coupling Parameters of the Faulty IM via Sparse Identification and Trigonometric Interpolation Polynomial

The main issue to address in this work is the accurate computation of the coupling parameters to develop a faulty IM analytical model minimizing computational effort and simulation times. These inductance or coupling parameters matrix should be calculated depending on the rotor position and saved in a Simulink 3D look up table, where the third dimension corresponds to the rotor position. This computation is performed offline using FEM, whose accuracy is widely accepted. However, this method has several drawbacks that the proposed method tries to overcome, as illustrated in the following subsections.

### 3.1. Computation of the Coupling Parameters Using FEM

**Figure 2:**
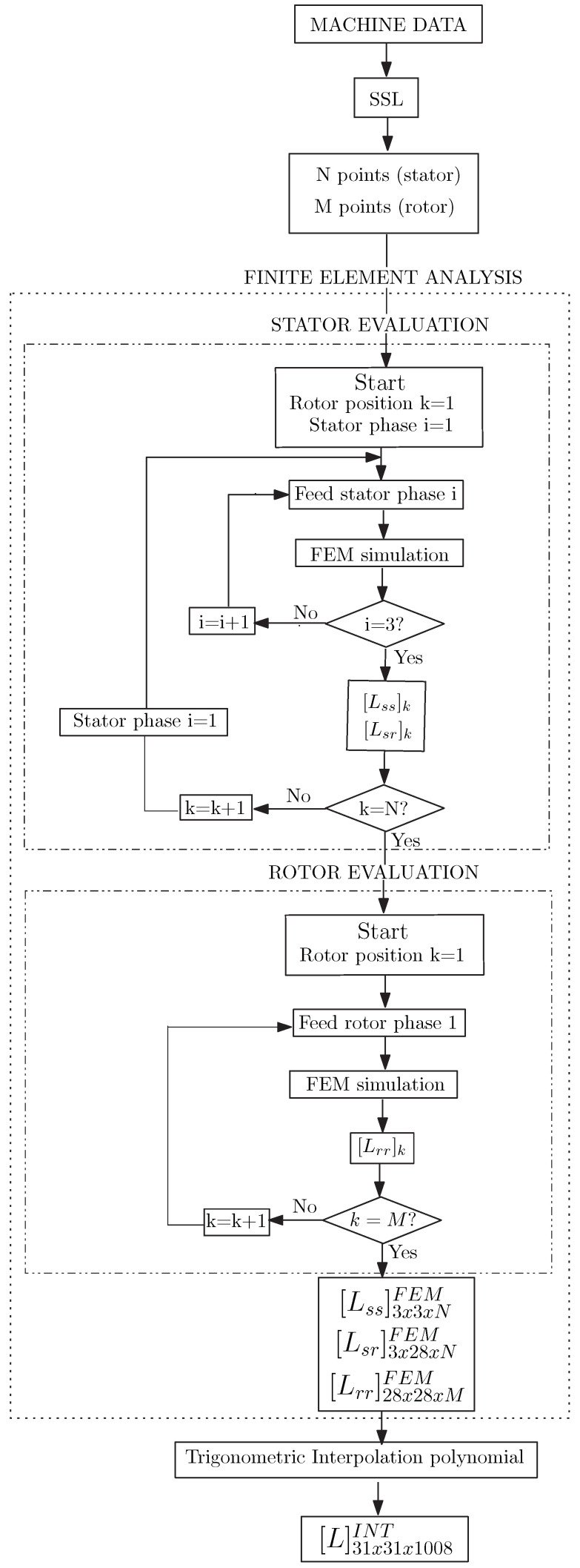
Diagram of the proposed method to obtain the inductance matrix of an IM model using FEM-based software and sparse subspace combined to trigonometric interpolation polynomial techniques.

The general process to compute the coupling parameters $\left[L\right]$ using FEM can be followed within the finite element analysis section of the diagram in Figure 2. First, the FEM-based model is built according to the geometry of the machine and the characteristic of the fault. For each rotor position, each stator phase is fed with 1A of direct current and the magneto-static FEM simulation runs. The results obtained allows calculation of the coupling parameters between stator phases $\left[L_{ss}\right]$ and between stator and rotor phases $\left[L_{sr}\right]$ for a given rotor position *N*. After that, each rotor phase is also fed with 1A of direct current, the FEM magneto-static simulation runs again to obtain the parameters $\left[L_{rr}\right]$ for the corresponding rotor position *M*. As a result, a three dimension coupling parameters matrix $\left[L\right]$ is obtained, whose first and second dimension correspond to the inductance related to the stator and rotor phases and the third to the rotor position. Therefore, the higher the number of rotor positions, i.e., the smaller the movement of the rotor for each step, the higher the accuracy and position resolution of the coupling parameters matrix $\left[L\right]$. Likewise, greater accuracy involves longer running times, and higher requirements for computing power and memory resources.

The process just described assumes linear conditions for the computation of the coupling parameters of the faulty IM. Magnetic effects such as saturation have little effect of fault harmonics and the main objective of this work is to present an efficient method for computing the coupling parameters of an IM that reproduces accurately the effect of the static eccentricity. Hence, considering only the linear, incremental problem, the results are less computational intense and a reasonably accurate solution for fault diagnosis purposes.

In this paper, a different way of addressing the computation of the inductance matrix is undertaken, presenting a new method based on FEM results but a much lower cost, while keeping good accuracy. FEM is used to compute the coupling parameters for a few specific rotor positions. Once these FEM coupling parameters are computed, they are used to build a trigonometric interpolation polynomial basis from which the coupling parameters for the other rotor positions are obtained.

### 3.2. Case of Study

The proposed method is applied to an IM whose characteristics are shown in Table 1, focusing on static eccentricity fault case, which is one of the most common mechanical faults in IMs [34]. Static eccentricity fault occurs when the axis of rotation coincides with the axis of rotor, but it displaces from axis of stator [35]. The positions for the minimum and maximum air-gap widths are fixed regarding the stator for any rotor orientation.

The severity of the fault is usually defined by degrees, between 0% for healthy machine (axis of rotation coincides with the axis of rotor and the axis of stator) and 100% for the maximum rotor rotation center displacement, which corresponds to the maximum displacement of the rotor rotation, 0.28 mm in the case of study.

On the other hand, to exemplify the cost savings in running times, computer power and memory resources, the inductance matrix for each degree of fault severity was obtained using FEM software open source femm 4.2 running on a computer with intel processor (R) Core (TM) i5-6400 CPU@2.70 GHz and 16 GB of RAM memory. To build the FEM model, as aforementioned in the previous subsection, it is necessary to feed sequentially the stator phases (*i* in Figure 2), a rotor phase, perform the magneto-static analysis and compute the inductances for each rotor position. Regarding the rotor positions (*k* in Figure 2), a total number of 28×36=1008 positions have been considered, which is the result of multiplying the number of stator slots by the number of rotor bars, K=RotorBars·StatorSlots. Therefore, for each rotor position, the rotor moves in increments of rd=2π/k=2π/1008=0.00632 rads. Each FEM simulation lasts about 1 min and takes up 22.5 MB of disk. For a generic scenario, considering 1008 rotor positions, a rotor phase is the loop (44) of two adjacent rotor bars (28), and the stator phases (3), each fault severity needs a total of (14+3)×1008 = 17,136 FEM simulations, which means approximately 12 days and more than 370 GB of memory space for saving the results.

Specifically in the case of static eccentricity fault, it is possible to reduce the number of simulations taking into account the symmetry of the machine. When a stator phase is fed, each rotor bar has the same flux linkage but with a specific geometry offset. According to this, the rotor positions can be reduced to a rotor bar travelling through a stator slot (N=36 in Figure 2) to calculate the coupling parameters between stator and stator phases $\left[L_{ss}\right]$ and between stator and rotor phases or bars $\left[L_{sr}\right]$. On the other hand, only the feeding of one rotor phase along a half of the rotor positions (M=504 in Figure 2) is required to obtain the coupling parameters between rotor bars $\left[L_{rr}\right]$. Therefore, to identify the coupling parameters of one machine working under a certain static eccentricity degree it needs 3×36+504=612 FEM simulations, which implies a computing time of 10.2 h and 13.45 GB of memory. It represents a significant reduction of both simulation times and computing effort, but these values are for machine and with a single severity degree of a given fault. Testing fault diagnosis techniques to be implemented in embedded devices, as well as the training of expert systems to classify faults, involve a considerable number of machines and fault scenarios. As a result, time and computing requirements continue to be excessive, whereby there is a rising interest in approaches that try to reduce these requirements.

### 3.3. Proposed Method Based on Sparse Identification and Trigonometric Interpolation Polynomial to Compute the Coupling Parameters under Static Eccentricity Conditions

Alternatively, ref. [30] apply the SSL to compute the inductance matrix for each desired degree of failure, based on the values obtained from FEM simulations. However, it still requires many FEM simulations, since the parameterization of a new faulty IM as well as every fault and every severity degree of the same fault involve the input of several inductance matrices fully FEM computed with their corresponding long simulation times and memory requirements. In fact, in [30] the polynomial basis is obtained with the data of 9 models fully computed with FEM which means more than 90 h of simulation time and more than 120 GB needed to save the results.

In a try to address these drawbacks, this paper proposes the use of the sparse identification and trigonometric interpolation polynomial to compute the coupling parameters matrix $\left[L\right]$ of an IM under static eccentricity conditions. From a small number of FEM simulations set via the sparse identification, a trigonometric polynomial basis is built to obtain the coupling parameters matrix. Following the same reasoning this method could be extended to other types of faults or even simultaneous faults, since the algorithm for computing the inductance matrix of the IM will compute the suitable terms to faithfully reproduce each type of fault. In the case of end ring-related faults can be simulated with the proposed model using simple tensor transformations from the starting resistance and inductance matrix, following the procedure proposed in [36].

Hence, the process will reduce computing time and memory requirements while keeping a similar accuracy to FEM with the interpolated solution using the trigonometric approximation.

**Figure 3:**
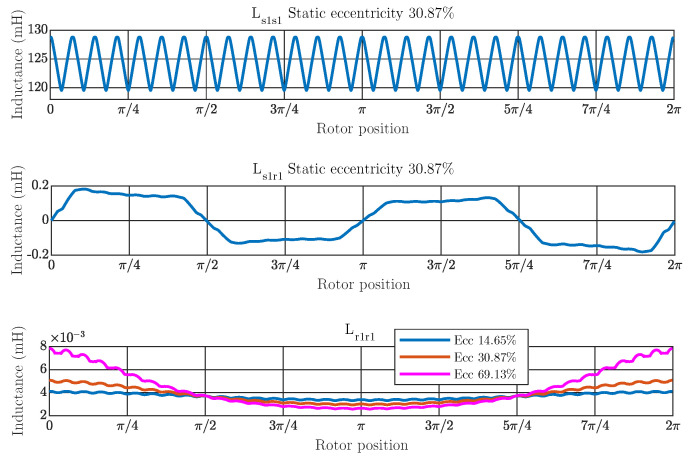
Coupling parameters between stator phase 1 and itself (**top**), between stator phase 1 and rotor bar 1 (**middle**) and between rotor bar 1 and itself (**bottom**) for static eccentricity of 30.87% depending of the rotor position using FEM simulations. The bottom graph also shows the coupling parameters between rotor bar 1 and itself for static eccentricity of 14.65% and 69.13%.

As the coupling parameters or inductance matrix of the IM model under a specific degree of eccentricity fault change with the rotor stepping, this paper proposes the SSL to select the rotor positions θ in the parametric space $\left[\theta_{min},\theta_{max}\right]$ in which perform the FEM simulations. Once the coupling parameters for these specific rotor positions have been computed via FEM, they are used to build the trigonometric polynomial basis [37]. This basis allows reliable calculation of the parameters of the inductance matrix $\left[L\right]$ for the remaining rotor positions. To determine the parametric space, it must be underlined the geometry characteristics of the IMs under static eccentricity fault. To illustrate these characteristics, Figure 3 shows the coupling parameters between the stator phase 1 and itself $\left[L_{s1s1}\right]$, between stator phase 1 and rotor bar 1 $\left[L_{s1r1}\right]$ and between rotor bar 1 and itself $\left[L_{r1r1}\right]$ for a static eccentricity of 30.87% depending on the rotor position using FEM simulations. As can be noted from the graphs, they are periodic functions. Therefore, a trigonometric polynomial approach can improve the interpolation performance to compute the coupling parameters when compared to other approaches based on algebraic polynomials such as Lagrange interpolation [38].

The bottom graph of the Figure 3 presents additionally the characteristics of the coupling parameters between rotor bar 1 and itself $\left[L_{r1r1}\right]$ for a static eccentricity of 14.65% and 69.13%. This graph illustrates that according to the degree of failure, the coupling parameters between rotor bars approximates to a 2π period function, and the higher the degree of eccentricity, the greater the amplitude of the function. Furthermore, the slot effect causes a ripple, which is associated with the movement of a rotor bar through a stator slot. Apart from this, the trigonometric polynomial basis should include the same space harmonics contents of the inductance matrix as the space harmonics of the inductance matrix obtained using FEM, as will be discussed in the next section.

For the case of trigonometric interpolation polynomial, the recommendations are to select equally spaced points in the parametric space [38]. Thus, Table 2 presents the selected rotor positions θ for $\left[L_{ss}\right]$ and $\left[L_{sr}\right]$ in the parametric space $\left[0,\pi /14\right]$ where the FEM simulations are performed. The parametric space of the coupling parameters $\left[L_{ss}\right]$ and $\left[L_{sr}\right]$, only requires the bar travelling a stator slot, 36 rotor positions for computing their values in the complete parametric space, in other words, it is only necessary the rotor movement from 0 to π/14 rad. A new rotor position of the parametric space interval is added to the set of points as long as the results of this FEM simulation significantly improve the accuracy of the computed new interpolation basis. It will notably reduce the number of FEM simulations and therefore, the computing requirements, while keeping a good accuracy of the coupling parameters of the faulty machine model.

On the other hand, the mutual inductance between rotor bars, due to their geometric complexity, requires a detailed explanation of their parametric space to interpolate as well as the selected points to perform FEM simulations. To reproduce accurately both the eccentricity and slot effect characteristic of $\left[L_{rr}\right]$, 5 points of each parametric subspace for a rotor slot (every 36 rotor positions from 0 to π) are selected to perform the FEM simulations. Thus, the subsequent trigonometric interpolation polynomial only have 5 space harmonics and moreover the amplitude is readjusted to the $\left[L_{rr}\right]$ function, reducing the fully FEM simulations to on ninth.

Once the set FEM simulations are performed, the parametric basis is developed using the trigonometric interpolation polynomial with separated cases for odd and even number of data [39]. For an odd number of nodes ($p^{\left(k\right)}=2m+1$), traditional trigonometric interpolation polynomial has the form:

(13) $$L_{ab}^{2m+1}\left(\theta \right)=\frac{c_{0}}{2}+\sum_{i=1}^{m}\left(c_{i}\;cos\;\left(i\theta \right)+d_{i}\;sin\;\left(i\theta \right)\right)$$

For an even number of data ($p^{\left(k\right)}=2m$) traditional trigonometric interpolation polynomial has the form:

(14) $$L_{ab}^{2m}\left(\theta \right)=\frac{c_{0}}{2}+\sum_{i=1}^{m-1}(c_{i}\;cos\;\left(i\theta )+d_{i}\;sin\;\left(i\theta \right)\right)+\frac{a_{m}}{2}\;cos\;\left(m\theta \right)$$

The space harmonics of the coupling parameters computed could introduce cross terms that would have a major impact in the fault diagnosis results [40]. Specifically, these cross terms would greatly affect the current simulated results and their harmonic content. For this reason, this work proposes a small modification of the Expressions (13) and (14), considering the space harmonics in the computation of the coupling parameters. For that purpose, the exponent *i* of the expressions does not vary from 1 to *m* in 1 to 1, but it does according to the harmonic content of $\left[L_{ss}\right]$, $\left[L_{sr}\right]$ and $\left[L_{rr}\right]$, respectively. Thus, in the case of stator self-inductance, the space harmonics take values from 28 in 28, because of the influence of the 28 rotor slots. This number 28 corresponds to the rotor bars of the machine simulated, as can be seen in Table 1. Likewise, in the case of rotor self-inductance the space harmonics take values from 36 in 36, because of the influence of the 36 stator slots.

Therefore, the trigonometric interpolation is applied to obtain the polynomial basis with which computes the inductance matrix. The base is generated for the whole range of rotor positions, obtaining a 2D matrix for every rotor position. Therefore, only the coefficients of the polynomial are saved, which reduces the memory requirements compared to other approaches where all the positions must be preset, directly obtaining a 3D inductance matrix instead.

The proposed method can be summarized in the following steps, which are illustrated in Figure 2:

1. Define the parametric space of the fault (Section 3.3).

2. Create the set of equally spaced points (Section 3.3).

3. Calculate the inductance matrix for the set of points obtained in step 2 using FEM simulations and following the process described in Section 3.1.

4. Develop the trigonometric polynomial basis from the results of step 3 using Equations (13) and (14).

5. Calculate the inductance matrix for the desired degree of severity of the fault using the polynomial base obtained in the step 4. It must be highlighted that contrary to analytical approaches as modified winding function approach (MWFA)-based models where the trigonometric interpolation is used to model the air gap and simplifications as radial magnetic field *B* in which the magnetic permeability is infinite and smooth air gap are assumed [41], in this work the full motor geometry and material are modeled through FEM, which considers the actual geometry of the air gap and the tangential component of the magnetic induction.

## 4. Results

**Figure 4:**
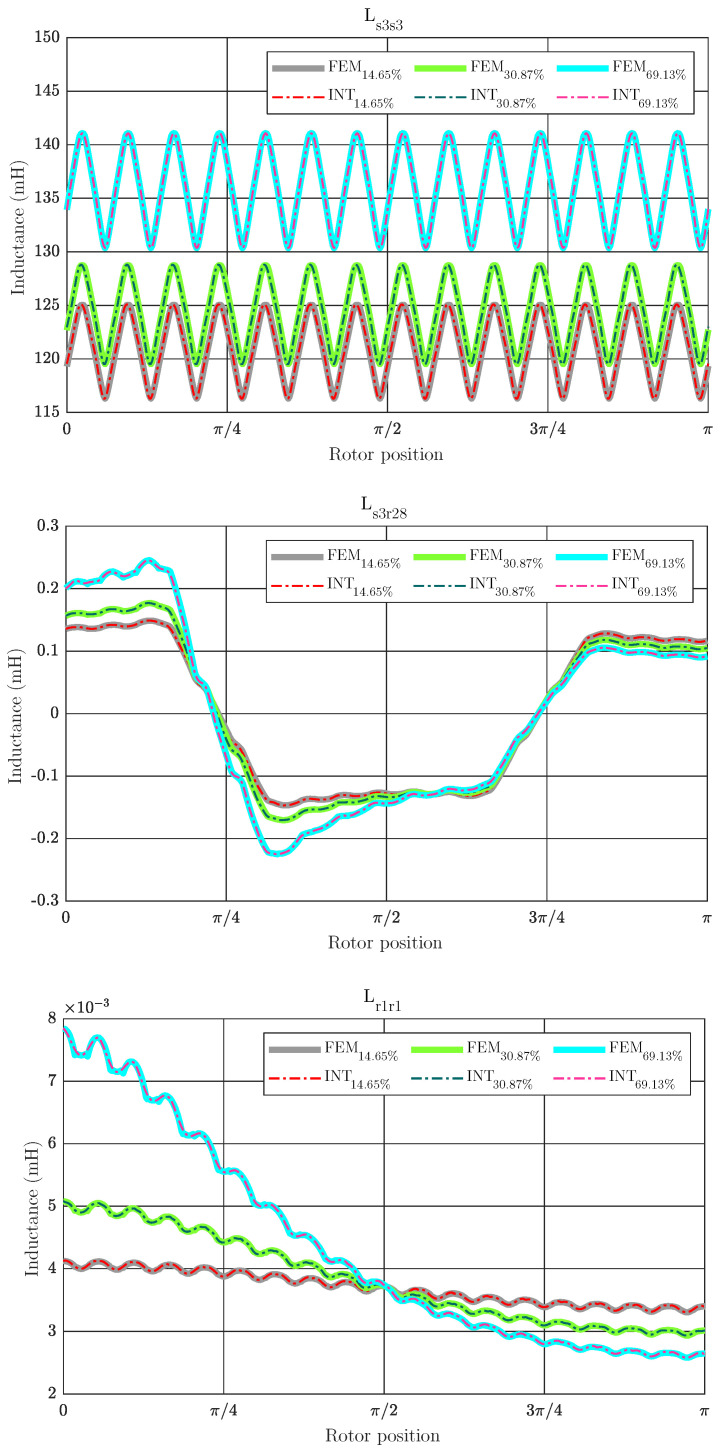
Coupling parameters between stator phase 3 and itself, between stator phase 3 and rotor bar 28 and between rotor bar 1 and itself depending on the rotor position using FEM simulations (FEM) and the proposed method (INT), for three different degrees of static eccentricity fault. The proposed method obtains mostly the same values as FEM.

To illustrate the accuracy of the proposed method, the inductance matrix for three different levels of static eccentricity (14.64%, 30.87% and 69.13%) are computed and compared with those obtained with a full FEM analysis as shown in Figure 4, for the coupling parameters between the stator phase 3 and itself, $\left[L_{s3s3}\right]$, between the stator phase 3 and rotor bar 28, $\left[L_{s3r28}\right]$, and between the rotor bar 1 and itself, $\left[L_{r1r1}\right]$ respectively. As can be seen, the coupling parameters obtained with the proposed method are essentially the same as those computed using only FEM, but minimizing the computational effort as shown in Table 3. This table illustrates the resulting computational savings for the proposed method, which computes the coupling parameters almost 10 times faster and only needs a 11% of the memory resources required when compared to the generic case particularized for a static eccentricity fault.

## 5. Fault Diagnosis Analysis

The inductance matrix obtained using FEM, sparse identification and trigonometric polynomial interpolation, $\left[L^{INT}\right]$, as well as the obtained inductance matrix using fully FEM simulations, $\left[L^{FEM}\right]$ are implemented in the analytical model shown in Figure 1. This model runs in real time in the HIL OP4500 from OPAL-RT, whose characteristics can be found in Appendix A. The eccentricity fault is detected using the motor current signature analysis (MCSA) method so that the different centers of rotor and stator axes result in current harmonic components induced in the stator winding at frequencies calculated through the expression [42]:

(15) $$f_{h}=\left[\left(k\cdot R_{d}\pm n_{d}\right)\frac{1-s}{p}\pm \nu \right]\cdot f_{1}$$

where *k* is any positive integer, $n_{d}$ is 0 for static eccentricity case, *s* is the slip, *p* is the number of pole pairs, $R_{d}$ is the number of rotor slots, *f* is the main frequency and ν is the order of the stator time harmonics. According to specific machine parameters defined in Table 1, $R_{d}=28$ and p=2.

**Figure 5:**
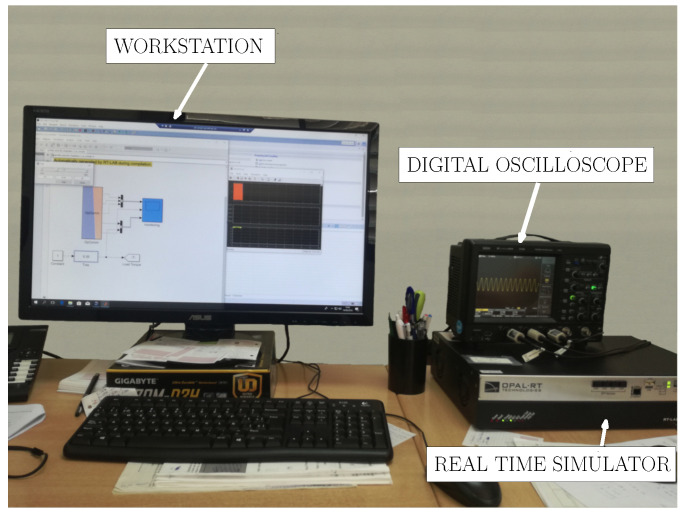
Faulty hybrid FEM-Analytical model implemented in HIL System OP4500. Stator currents are acquired using a digital oscilloscope properly connected to the HIL. HIL characteristics are found in Appendix A.

The stator current signals are acquired using the analog outputs of the HIL device, as shown in Figure 5. These current samples can be used for fault diagnosis and classification under different working conditions, the development and training of expert fault diagnosis systems, the creation of data bases, etc.

### 5.1. Detection of the Fault Harmonics under Transient Conditions

It is very common for IMs to work under transient conditions, such as the start-up transient, varying load conditions or supplying frequency changes. In these cases, the Expression (15) indicates that the frequency of the fault harmonics is no longer constant, but changes with the motor slip.

Under these conditions, traditional fast Fourier transform (FFT)-based diagnostic techniques cannot be used for fault diagnosis purposes. The current analysis of the start-up transient of the IM and time–frequency distributions as the spectrogram [10], can correctly detect and generate the evolution of the fault harmonics in the joint time–frequency domain [43].

This approach has attracted a rising interest in the technical literature in recent years. It provides advantages such as greater accuracy, since various operating points are analyzed. On the other hand, the slip evolution is well known, from 1 to ≃0 in start-up transient conditions, which allow identification of the patterns in the fault harmonic components. Moreover, IMs such as wind turbines usually work under non-stationary working conditions, so developing diagnosis techniques and systems that can work under these conditions is reasonable. Thus, the amplitude evolution of the upper side harmonic (USH) is used to validate the proposed model in transient regime, using the start transient of the IM. The fault analysis is based on Gabor analysis of the current to capture the characteristic pattern from the start-up machine current generating an image of the trajectory of the USHst in the time–frequency plane. The procedure followed is detailed in [44]. The method is mainly based on the development of Gaussian window to capture the transient of the fault harmonic USH, which is computed as:

(16) $$g\left(t\right)={\left(2\beta \right)}^{1/4}\exp-\beta \pi t^{2}$$

where β is theoretical slope in hertz per second of the fault harmonics in the time–frequency plane, defined as $\beta =\Delta f/t_{startup}$, Δf being the variation of the fault harmonic frequency and $t_{startup}$ the duration of the transient.

**Figure 6:**
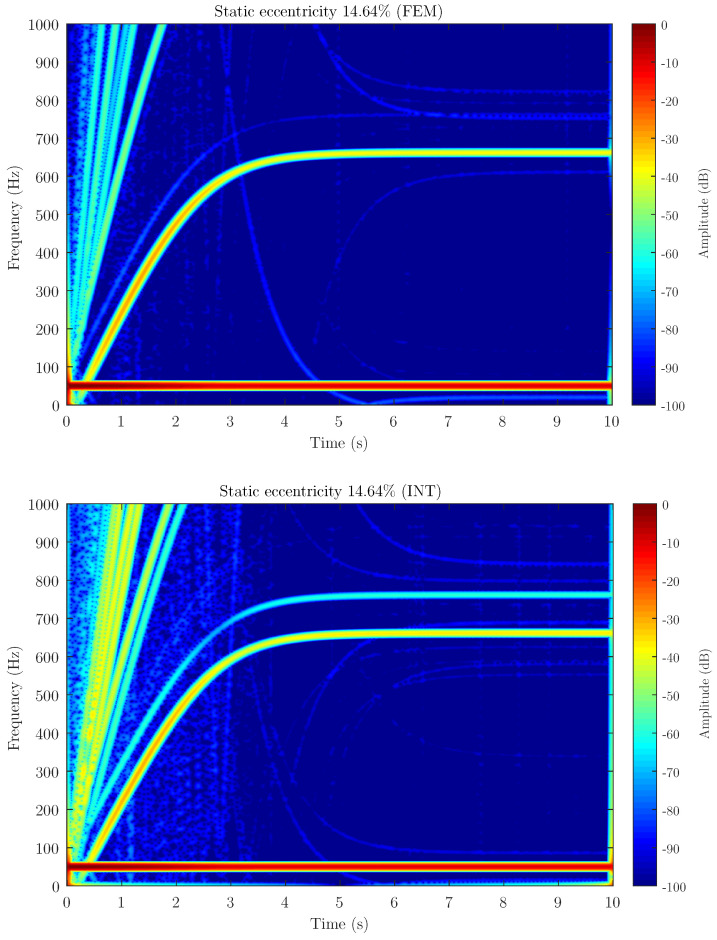
Stator current spectrogram for static eccentricity level 14.64% using FEM software (FEM) and the proposed method (INT) to calculate the coupling parameters of the hybrid FEM-Analytical model. The hybrid model obtains a good approximation of the amplitude for detecting the presence of the fault.

**Figure 7:**
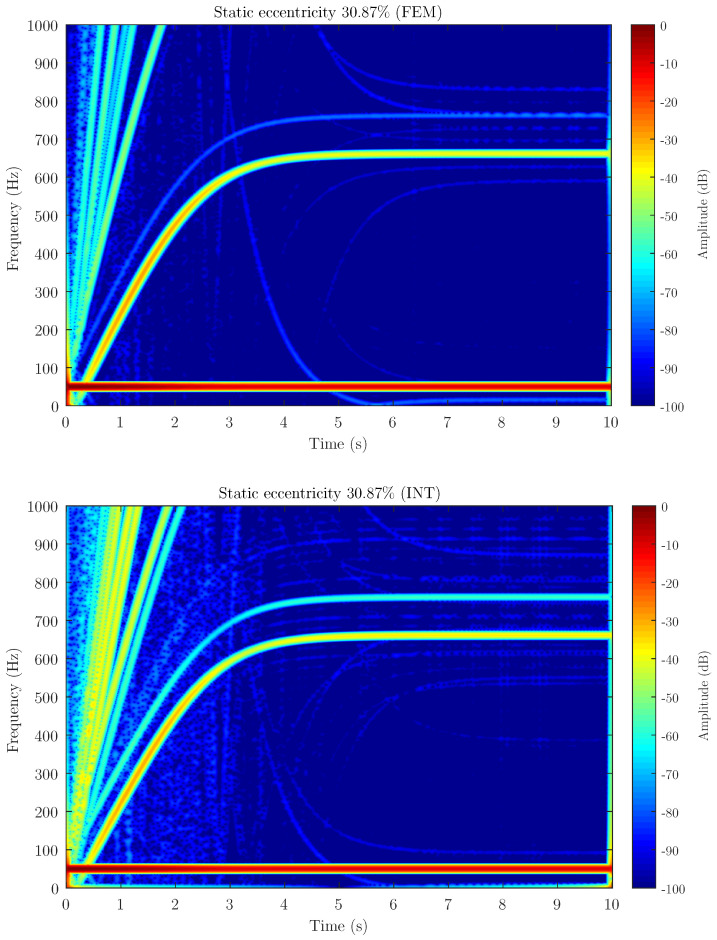
Stator current spectrogram for static eccentricity level 30.87% using FEM software (FEM) and the proposed method (INT) to calculate the coupling parameters of the hybrid FEM-Analytical model. The hybrid model obtains a good approximation of the amplitude for detecting the presence of the fault.

**Figure 8:**
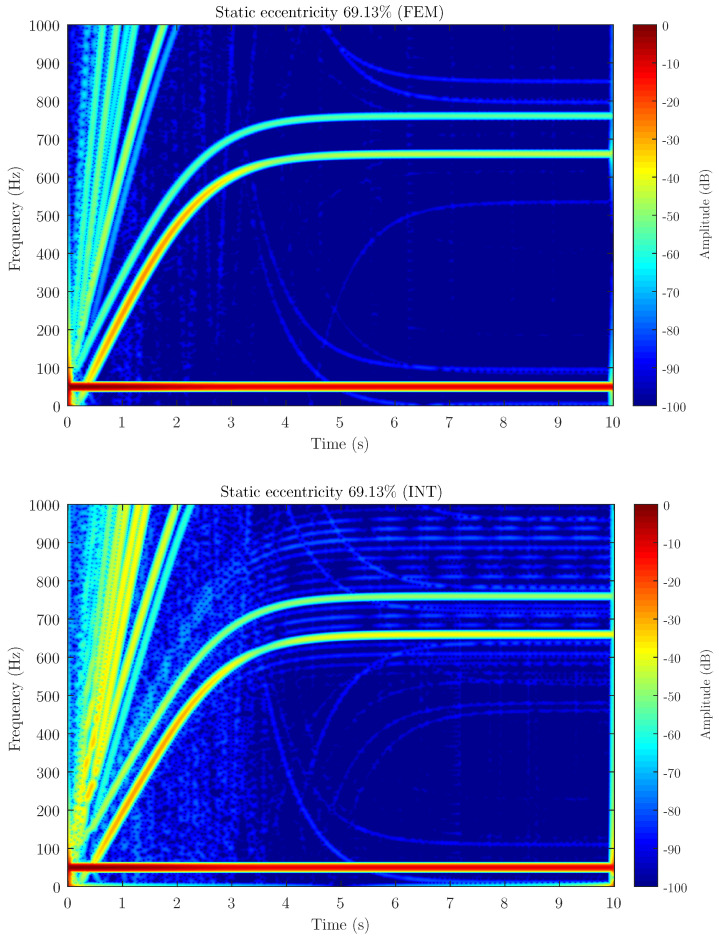
Stator current spectrogram for static eccentricity level 69.13% using FEM software (FEM) and the proposed method (INT) to calculate the coupling parameters of the hybrid FEM-analytical model. The hybrid model obtains a good approximation of the amplitude for detecting the presence of the fault.

Figure 6, Figure 7 and Figure 8 show the spectrogram of the start-up stator current of the simulated machine using the coupling parameters computed via full FEM analysis (FEM in the figures) and with the proposed method (INT in the figures) for the three different levels of static eccentricity 14.64%, 30.87% and 69.13% respectively. As can be seen, the proposed model is able to display the characteristic signature of the static eccentricity fault. Moreover, these figures illustrate that the amplitude of the USH signature is greater as the fault severity degree increases when using both FEM and the proposed method. Therefore, the proposed method could be a very good approximation for fault diagnosis purposes.

### 5.2. Effect of Space Harmonics into the Fault Analysis under Transient Conditions

**Figure 9:**
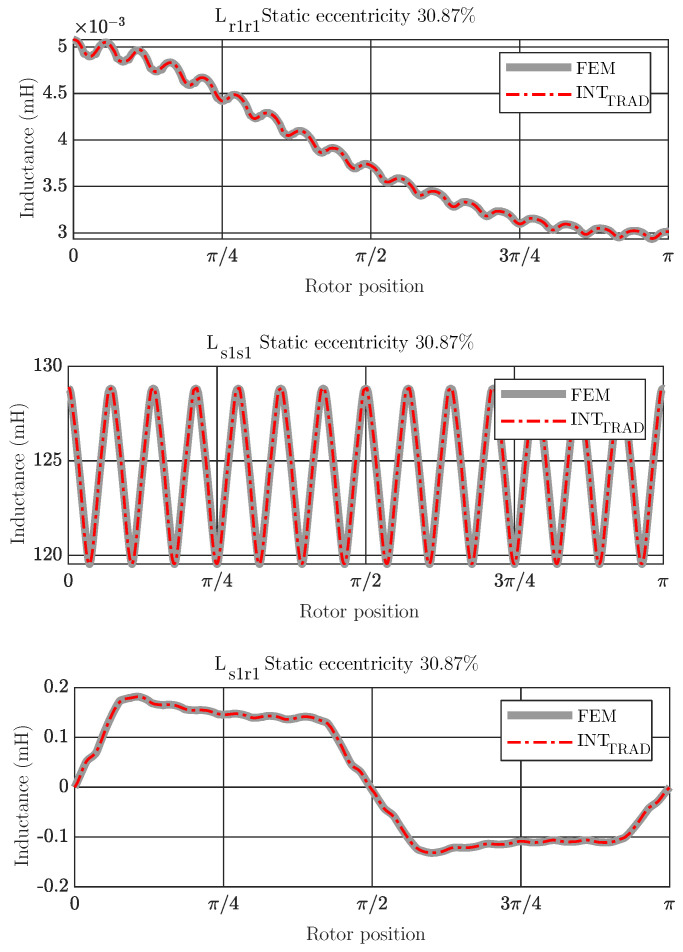
Coupling parameters between rotor bar 1 and itself (**top**), between stator phase 1 and itself (**middle**) and between stator phase 1 and rotor bar 1 (**bottom**), depending on the rotor position using FEM simulations and traditional trigonometric interpolation polynomial for 30.87% of static eccentricity. Both methods obtain essentially the same values as FEM.

The effect of the spaces harmonics when applied the trigonometric interpolation polynomial to build the basis to obtain the coupling parameters is analyzed as follows. The inductance matrix for a level of static eccentricity of 30.87% is computed using trigonometric interpolation and compared with the inductance matrix obtained with a full FEM analysis, as shown in Figure 9, for the coupling parameters between the stator phase 1 and itself, $\left[L_{s1s1}\right]$, between the stator phase 1 and rotor bar 1, $\left[L_{s1r1}\right]$, and between the rotor bar 1 and itself, $\left[L_{r1r1}\right]$ respectively.

As can be seen, the coupling parameters obtained with the trigonometric interpolation are essentially the same as those computed using only FEM, and therefore very similar to the results obtained using the approach considering the space harmonic content.

**Figure 10:**
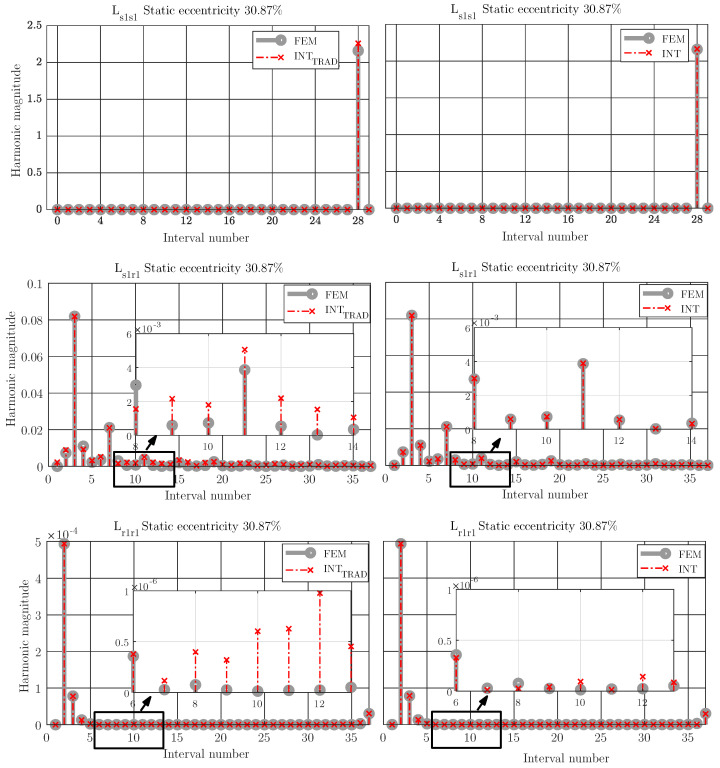
Space harmonics content of the coupling parameters between stator phase 1 and itself (**top**), between stator phase 1 and rotor bar 1 (**middle**) and between stator phase 1 itself (**bottom**), in the case of 30.87% of static eccentricity using FEM simulations and traditional trigonometric interpolation polynomial (**left**) and using FEM and trigonometric interpolation considering space harmonics (right). The traditional trigonometric interpolation obtains values of space harmonics other than FEM while both the order and the amplitude of the space harmonics contents using the proposed method are the same as using FEM with a very small error.

Regarding the space harmonics, Figure 10 on the left shows the space harmonic content of the coupling parameters between stator phase 1 and itself, stator phase 1 and rotor bar 1 and between the rotor bar 1 and itself in the case of 30.87% of static eccentricity, using the trigonometric interpolation polynomial and trigonometric interpolation polynomial considering the space harmonics. The space harmonics obtained with trigonometric interpolation are considerably different to those obtained with FEM. Nonetheless, Figure 10 on the right shows the space harmonic content of the coupling parameters between stator phase 1 and itself, stator phase 1 and rotor bar 1 and between the rotor bar 1 and itself in the case of 30.87% of static eccentricity using trigonometric interpolation polynomial considering the space harmonics and compared to the obtained using FEM. It can be seen that the proposed method obtains good results in terms of space harmonic contents not only in order but also in amplitude compared with those computed with full FEM analysis.

**Figure 11:**
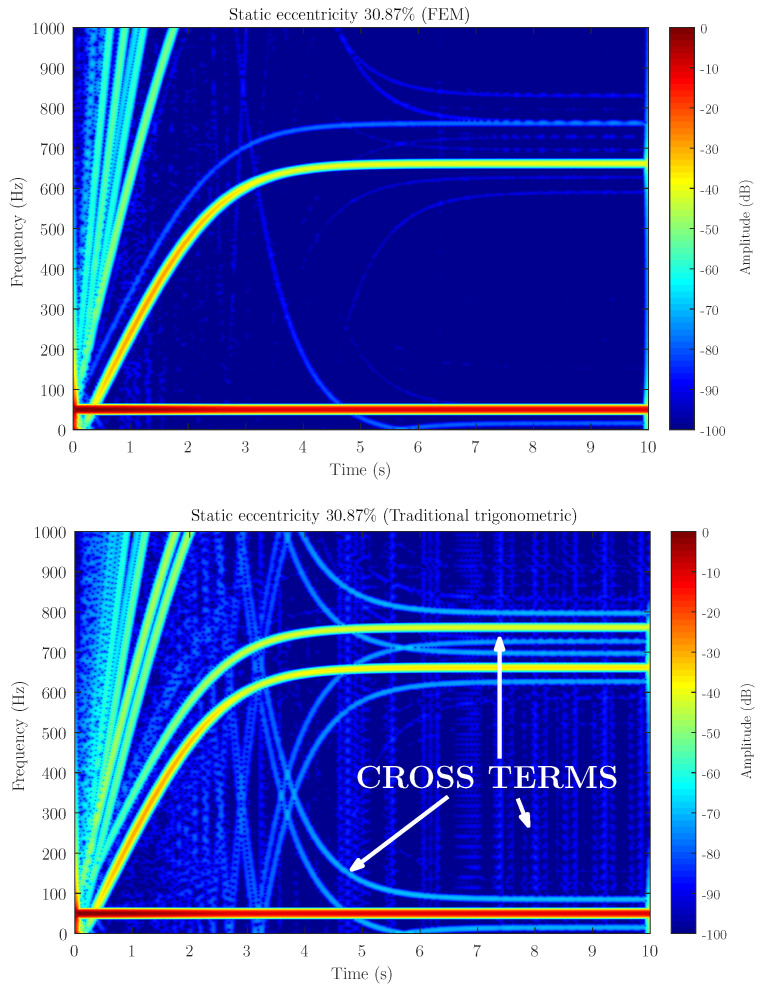
Stator current spectrogram for static eccentricity level 30.87% using FEM (**top**) and traditional trigonometric interpolation polynomial (**bottom**). Traditional trigonometric interpolation polynomial introduces cross terms in the post-processing that could lead to misdiagnosis.

Therefore, the ideal is that the parameters computed with the proposed method have not only the same distribution along the rotor position as shown in Figure 4 but also have the same content of space harmonics as shown on the right of Figure 10, as the coupling parameters computed with full analysis. The space harmonics of the coupling parameters based on trigonometric interpolation could introduce cross terms that would interfere in the fault diagnosis results [40]. These cross terms would greatly affect the current simulated results and their harmonic content, making the post-processing results difficult to interpret and not very accurate to the actual machine. Figure 11 illustrates the effect of considering the space harmonics to build the trigonometric polynomial basis. This figure shows the spectrogram of the start-up stator current of the simulated machine using the coupling parameters computed via FEM (top) and traditional trigonometric interpolation polynomial (Equations (13) and (14)) (bottom). As can be seen, the traditional method could make fault analysis difficult to interpret and lead to misdiagnoses.

Cross terms may even overlap with the desired harmonic signals. Thus, if the graphs of this figure are compared, it can be seen that the USH amplitude resulting using the traditional trigonometric interpolation polynomial is far from the USH amplitude obtained using FEM for the degree of severity simulated. The results obtained using the traditional trigonometric interpolation correspond to a higher degree of fault, which would imply a misdiagnosis.

Attending to the results, as can be seen in Figure 6, Figure 7 and Figure 8 when compared to Figure 11, the proposed method, which considers the space harmonic content, greatly reduces the cross terms that could interfere in the fault diagnosis analysis and obtains essentially the same results than FEM. Moreover, Figure 6, Figure 7 and Figure 8 also illustrates how the USH amplitude evolves according to the degree of eccentricity. The proposed method provides more accurate results than the traditional method, reproducing a very good approximation of the USH characteristic amplitude for the specific degree of eccentricity if compared to FEM method. Therefore, the proposed method, by obtaining more accurate results, could be used both for detecting the presence of the fault and establishing fault thresholds.

## 6. Experimental Validation

To validate the proposed method, an experimental setup has been arranged with a commercial 1.1 kW, 50 Hz IM, in healthy state and with eccentricity to compare the presence of the fault harmonics to the obtained results using the hybrid model. The characteristics of the machines used are given in Table 1. To achieve longer startup transients the IM has been feed to reduced voltage using an autotransformer and no external load.

**Figure 12:**
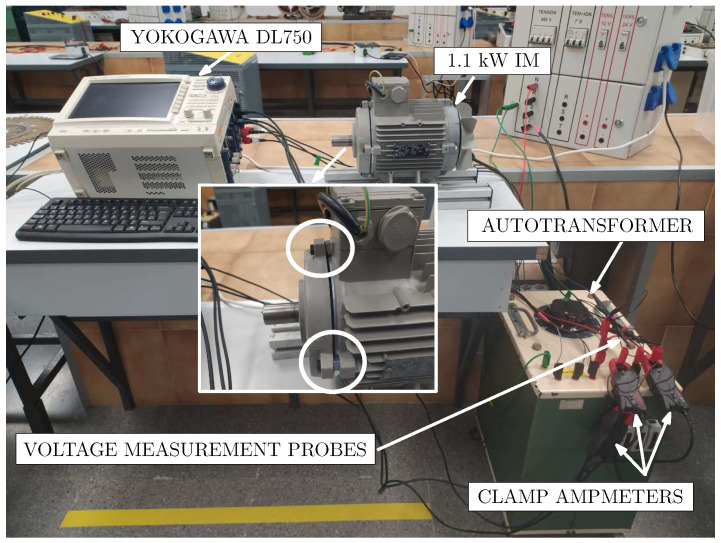
Experimental setup used for validation of the methodology. The zoom shows the IM hood fastener holes drilled to allow for static eccentricity faults.

**Figure 13:**
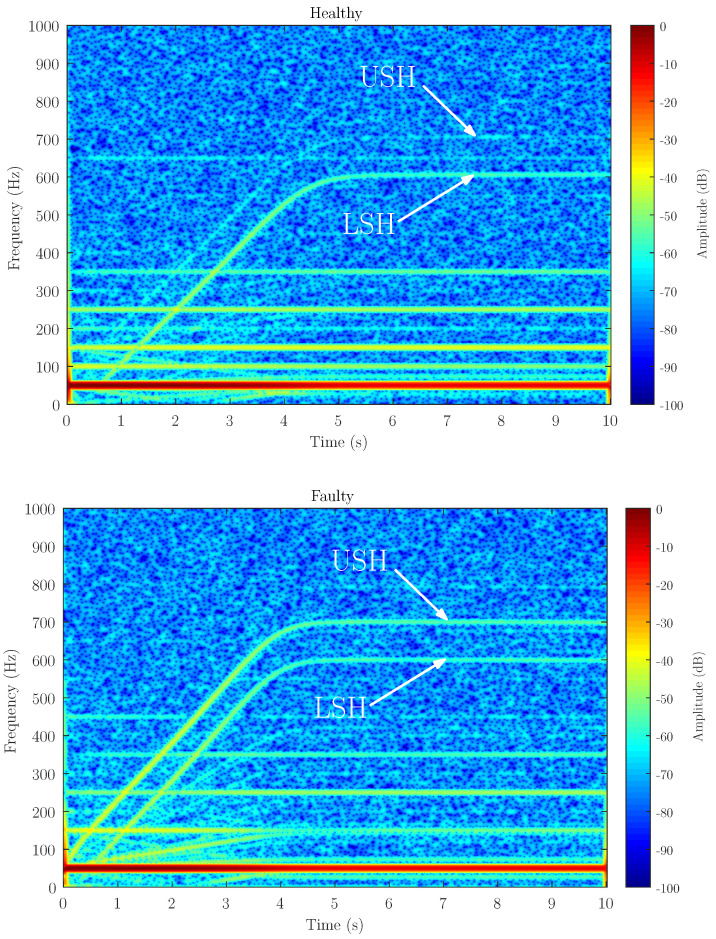
Stator current spectrogram of the experimental machine, in healthy (**top**) and in faulty (**bottom**) conditions. It can be seen the characteristic trajectory in the time–frequency plane of the USH, generated in the case of a start-up transient of 5 s, as well as the evolution of the USH amplitude, which increases as the degree of the severity fault does.

Secondly, to reproduce the eccentricity conditions, the hood fastening holes have been enlarged to introduce a small tolerance in the rotor axis place, as detailed in the zoom in Figure 12. This figure shows the experimental setup performed for the validation of the proposed method. The stator currents have been acquired using the current clamps connected to a Yokogawa DL750 Oscilloscope at a sampling rate of 10 kHz during 10 s. The stator current spectrogram of the actual machine in healthy or initial conditions (top) and in faulty conditions (bottom) is shown in Figure 13. As can be seen, the machine shows the same characteristic signature of the fault harmonic as the simulated machine. In addition, it should be noted that as well as using the proposed model, the amplitude of the USH increases when the fault degree does (Faulty), which confirm the simulation results. Magnetic saturation as well as other magnetic effects could affect to the space harmonics. However, these harmonics have little influence on the evolution and amplitude of the fault harmonics as can be seen if compared the results in Figure 7 and Figure 13. Therefore, this kind of analysis could be a very useful tool for condition-monitoring and fault diagnosis purposes in IMs.

## 7. Conclusions

Hybrid FEM-Analytical modeling has become a powerful tool for rotating electrical machine analysis, since it can provide very accurate modeling for various faulty IM conditions, offering insight and the needed signals for further analysis using signal processing and/or machine learning. By exploiting the benefits of this hybrid approach and advanced numeric techniques for computation, in this paper the SSL and the trigonometric interpolation polynomial are proposed to reduce the computation requirements to calculate an accurate hybrid FEM-Analytical model of a faulty IM. FEM is used to compute a reduced set of coupling parameters along the rotor positions. These FEM coupling parameters are used to build a trigonometric interpolation polynomial through which the full set of coupling parameters of the machine are computed. The proposed method has been illustrated for various degrees of static eccentricity fault, from an incipient level to more severe, to associate the amplitude of the fault harmonic components with the fault severity degree. Following the same reasoning the method could be extended to other types of faults or even to simultaneous faults since the proposed algorithm will compute the suitable coefficients to faithfully reproduce each type of fault.

Coupling parameters are calculated in Section 3, and the results are saved in 3D matrices as a function of rotor position. Using the SSL and taking into account the symmetry characteristics of the fault, the parametric space and the simulation points for FEM are defined. Thus, the trigonometric polynomial basis is developed and the inductance matrix for the desired degree of severity computed. As shown in the paper the proposed method obtains a similar accuracy to fully FEM analysis to compute the coupling parameters of a faulty machine; however, the computing requirements are significantly smaller. Once the coupling parameters are calculated, they can be used in the analytical dynamic model where the currents can be investigated. The fault diagnosis results, i.e., fault harmonic components, have been compared to those obtained using fully FEM simulations to compute the coupling parameters. The method has been illustrated for the static eccentricity fault, but the same approach can be applied to other types of faults and/or different degrees of severity. For the validation of the results, the frequency spectrum of the stator current measured in a laboratory set up under healthy and eccentricity case is analyzed. Thus, the approach proposed can contribute to the development of the testing of fault diagnosis techniques to be implemented in embedded devices, as well as to train expert systems to assess the machine condition.

## Figures and Tables

**Table 1:** Data of the simulated machine.

**Power**	1.1 kW	**Pole pairs**	2
**Voltage**	230/400 V	**Speed**	1415 rpm
**Current**	4.4/2.55 A	**$N^{o}$ of rotor bars**	28
**Frequency**	50 Hz	**$N^{o}$ of stator slots**	36
**Air-gap length**	0.28 mm	**Type of fault**	Static Eccentricity

**Table 2:** Set of points in the parametric space $\left[0,\pi /14\right]$ to compute $\left[L_{ss}\right]$ and $\left[L_{sr}\right]$.

Point	Rotor Position θ (rad)
1	0
2	0.0374
3	0.0748
4	0.1122
5	0.1495
6	0.1867
7	0.2244

**Table 3:** Computational costs, computational time and memory resources to obtain the coupling parameters of a faulty IM for a generic case, a case of static eccentricity and using the proposed method.

	FEM	Computation	Memory
	Simulations	Time	Resources
Generic case	17,136	11 days 21 h 36 min	376.52 GB
Static eccentricity	612	10 h 12 min	13.45 GB
Proposed method	70	1 h 10 min	1.54 GB

## Appendix A. HIL OP4500 Main Features

Real-time target: 4 INTEL processor cores 3.3 GHz (only 1 activated).

Solid state disk: 125 Gb.

Memory RAM: 4 Gb.

Real-time operating system: Linux RedHat.

Xilinx Kintex 7 FPGA (326.000 Logic cells and 840 DSP slice).

Sampling rate: 200 MHz.

96 user inputs/outputs (I/O): 16 analog inouts and 16 analog outputs, 24 digital inputs and 24 digital outputs, 8 RS422 digital inputs and 8 RS422 digital outputs.
